# Evaluation of porogen factors for the preparation of ion imprinted polymer monoliths used in mercury removal

**DOI:** 10.1371/journal.pone.0195546

**Published:** 2018-04-12

**Authors:** Siti Khadijah Ab. Rahman, Nor Azah Yusof, Abdul Halim Abdullah, Faruq Mohammad, Azni Idris, Hamad A. Al-lohedan

**Affiliations:** 1 Chemistry Department, Faculty of Science, Universiti Putra Malaysia, Serdang, Selangor, Malaysia; 2 Institute of Advanced Technology, Universiti Putra Malaysia, Serdang, Selangor, Malaysia; 3 Surfactant Research, Department of Chemistry, College of Science, King Saud University, Riyadh, Kingdom of Saudi Arabia; 4 Chemical Engineering and Environmental Department, Faculty of Engineering, Universiti Putra Malaysia, Serdang, Selangor, Malaysia; Visvesvaraya National Institute of Technology, INDIA

## Abstract

In the present study, ion imprinted polymer monoliths (IIPMs) were developed to overcome the limitations of ion imprinted polymer particles (IIPPs) used for the removal of Hg(II) ions from waste water samples. The adsorbents preparation, characterization and Hg(II) removal were very well reported. The IIPMs on porogen optimization was prepared using the molding technique with Hg(II) as a template ion, [2-(methacryloyloxy)ethyl]trimethylammonium cysteine (MAETC) as ligand, methacrylic acid (MAA) as functional monomer, ethylene glycol dimethacrylamide (EGDMA) as cross-linker, benzoyl peroxide as an initiator and methanol and acetonitrile as porogen in the polypropylene tube (drinking straw) as mold. The IIPMs prepared with higher volumes of porogen were indicated to have a good adsorption rate for the Hg(II) removal along with good water permeability and larger porosity as compared to a lower volume of porogen. The IIPMs prepared using the binary porogen were able to improve the porosity and surface area of the monolithic polymers as compared to the single porogen added IIPMs. Finally, we indicate from our analysis that the IIPM having the efficient capacity for the Hg(II) ions is easy to prepare, and has higher water permeability along with high porosity and high adsorption capacity and all these factors making it one of the suitable adsorbent for the successful removal of Hg(II) ions.

## Introduction

Mercury (Hg) is one of the most toxic elements in the environment because of its high reactivity, extreme volatility and relative solubility in water and in living organisms [1]. The Hg contamination in the environment has unfortunately remained a serious problem despite the noticeable efforts in recent years [2]. The element is also famous for the fact that the ionic Hg and its derivatives tend to bioaccumulate in the human body which leads to the gathering of high concentrations of the element, which can cause kidney damage, neurological changes, paralysis, chromosome breakage, birth defects [3–4], brain damage [5] and gastrointestinal injure [2], to mention some. From this it is evident that the removal of Hg from drinking water and waste water is very important so as to protect the humans and the environment.

In recent years, ion imprinting technique has attracted enormous research interest as an adsorbent for the selective removal of different ions from aqueous samples. The high selectivity of ion imprinted polymers (IIPs) can be explained by the polymer memory effect towards the metal ion interaction with a specific ligand, coordination geometry, metal-ion coordination number, charge and size [6]. In general, the IIPs for the removal of Hg(II) can be prepared in particulate or powder form, which is the prepared ion imprinted polymer particle (IIPP) needs to be crushed, ground and sieved into fine particles before proceeded [7–10]. The IIPPs obtained are usually irregular in shape and size, with poor binding site accessibility for the target ions [8, 10–11]. Moreover, the IIPPs packed columns are not suitable to commercialize/apply to the industrial columns as this may increase the column pressure, lower the water transport and increase the risk of bubble formation. For these reasons, the ion imprinted polymer monoliths (IIPMs) can serve as alternatives to the IIPPs as they can easily overcome the limitations of applying to the industrial columns. In addition, the introduction of IIPMs will avoid the tedious grinding and sieving procedures as well as the problems of costly particle losses, particle inhomogeneity, and ion imprinted spots loss and tedious synthesis procedures [8, 12–13]. The IIPMs can be prepared using the “molding process” in a variety of physical forms (such as long and short columns, disks, mold, and fibers) to suit the final application and to maintain the better analytical performance rates [11].

In general during the preparation of IIPMs the selection of porogen is a more critical process as more attention needs to be paid in comparison to the traditional methods where the wrong selection of porogens may lead to the generation of final IIPM’s architecture to be less efficient [11]. Since the removal and rebinding of template ions from the IIPMs surface are mostly governed by their porosity and surface factors when applied for the adsorption process, and so, the incorporation of an ideal porogen during the synthesis creates enough space in the IIPMs that can eventually turns out to be pores after the completion of synthesis. However, the IIPMs prepared in the absence or lesser amounts of porogen are responsible for the generation of surface architectures which are consistently too firm, dense, and hardly can bind the guest ions [14].

In the present paper, we report the preparation of IIPMs using methacrylic acid (MAA) as a monomer, ethylene glycol dimethacrylate (EGDMA) as cross-linker, [2-(methacryloyloxy)ethyl]trimethylammonium cysteine (MAETC) as ligand for complexing the Hg(II) ions and methanol and acetonitrile as porogen in the polypropylene tube (drinking straw). A drinking straw was selected as a mold for the preparation of IIPMs as it is easy to get, cheap and stable even at high temperature conditions (melting point: 130–170°C). The IIPPs and various other types of IIPMs prepared were characterized using Fourier-transform infrared spectroscopy (FTIR), field emission scanning electron microscopy (FESEM), thermogravimetric analysis (TGA) and Brunauer-Emmett-Teller (BET) method. Further, the evaluation of porogen factors of the prepared IIPMs towards the removal of Hg(II) has been studied.

## Materials and methods

### Chemicals

A stock solution of 10 μg/L of Hg(II) aqueous solution was prepared by the dilution of 1000 mg/L of the standard of Hg(NO_3_)_2_ (Sigma-Aldrich) in deionized water (obtained with a Thermo scientific water system, Barnstead Nanopure). Sodium nitrate (NaNO_3_), potassium carbonate (K_2_CO_3_), ethyl acetate, thiourea, sodium hydroxide (NaOH), *L*-cysteine, ethylene glycol dimethacrylate (EGDMA) and [2-(methacryloyloxy)ethyl]trimethylammonium chloride (MAETC) and methacryloyl(oxyethyl)trimethyl ammonium chloride (MOTAC) were obtained from Sigma-Aldrich. 2-Hydroxyethyl methacrylate (HEMA) and methacrylic acid (MAA) were purchased from Fluka. Acetonitrile (CH_3_CN), benzoyl peroxide (BPO) and nitric acid (HNO_3_) were the products of R&M chemicals. Methanol (CH_3_OH), hydrochloric acid (HCl) and ethanol were obtained from J.T. Baker. Polypropylene tubes (drinking straw) were purchased from a shop (Seri Kembangan, Selangor, Malaysia).

### Preparation of IIPs

#### Synthesis of MAETC ligand

About 5 g of cysteine and 0.2 g NaNO_3_ were dissolved in 30 mL of K_2_CO_3_ (5%, w/v) aqueous solution and cooled to 0°C in the refrigerator. For this solution, 4 mL of MOTAC was added slowly under nitrogen atmosphere and stirred magnetically at room temperature for 3 h. After being stirred for the period, the solution was adjusted to a pH of 7.0 and was then extracted using ethylacetate and further the aqueous phase was evaporated in a rotary evaporator. The residue was crystallized in ethanol and ethyl acetate and the MAETC remained in the product was dried and preserved in a closed vial until further use.

#### Optimization of porogen factors for the preparation of IIPMs

The effect of different types and amounts of porogen in the IIPMs preparation using molding process was studied. First, 2 mmol of MAETC and 0.5 mmol of Hg(NO_3_)_2_ were taken in a 50 mL volumetric flask and made up to a total volume of 50 mL using methanol. Now, 5 mL of this solution was pipetted into a 100 mL beaker and 6 mmol of MAA, 3 mmol of HEMA, 30 mmol of EGDMA and 0.1 g of BPO were added under nitrogen atmosphere. After that, various amounts (5, 10, 20 and 30 mL) of porogens (CH_3_OH or CH_3_CN) were added into each IIPMs solution maintained under the nitrogen atmosphere and the detailed recipes are shown in Table 1. After the addition of porogen to the solution mixture under nitrogen atmosphere, the solution was stirred for 30 min, followed by the transferring of IIPMs solution into a drinking straw (diameter = 1.0 cm) as a mold. The solution loaded straws are then transferred to a water bath maintained at 70°C for 3 h and when the polymerization reaction gets completed, the IIPMs were cut into small pieces having 0.5 cm length and 1.0 cm diameter. The transferring of IIPMs solution to the drinking straw and further polymerization, cutting process can be performed even in the absence of nitrogen atmosphere. Also, for the preparation of IIPPs the same procedure was followed as similar to the IIPMs in terms of recipe and amounts of reagents, but the only difference is that the IIPP solution was polymerized in a beaker maintained under nitrogen atmosphere. After the completion of polymerization, the bulk polymer was broken and crushed into fine powder using a dry blender. The preparation of IIPPs and IIPMs was repeated by scaling up the amount of the reagents up to 200 times.

**Table 1:** Optimization of porogen factors for the preparation of IIPP and IIPMs.

Types of IIPs	Porogen (mL)		Metal-ligand complex in CH_3_OH solution (mL)	Monomer:Co-monomer:Cross-linker (ratio)	Initiator (g)
	CH_3_OH	CH_3_CN			
Single porogen (CH_3_OH)					
IIPP	-	-	5.0	2:1:10	0.1
IIPM-1	-	-	5.0	2:1:10	0.1
IIPM-2	5.0	-	5.0	2:1:10	0.1
IIPM-3	10.0	-	5.0	2:1:10	0.1
IIPM-4	20.0	-	5.0	2:1:10	0.1
IIPM-5	30.0	-	5.0	2:1:10	0.1
Binary porogen (mixture of methanol and acetonitrile)					
IIPM-6	-	5.0	5.0	2:1:10	0.1
IIPM-7	-	10.0	5.0	2:1:10	0.1
IIPM-8	-	20.0	5.0	2:1:10	0.1
IIPM-9	-	30.0	5.0	2:1:10	0.1

Metal-ligand (Hg(II)-MAETC) complex dissolved in methanol; 5mL.Monomer(MAA)-Co-monomer(HEMA)-Cross-linker(EGDMA) (ratio): 6.0:3.0:30.0 mmol; Initiator (BPO): 0.1 g.

The prepared IIPP and all of the IIPMs were washed with methanol/water solution (60/40, v/v, degassed) for several times in order to remove the unreacted monomers and other ingredients. Then, the polymers were washed with 0.5% thiourea in 0.05 M HCl solution for the removal of the template (Hg(II)) ions. This procedure was repeated several times until the Hg(II) ions could not be detected in the filtrate using the mercury analyser. The polymers were further cleaned by being incubated in 0.1 M of HNO_3_ for 3 h to further remove any residual Hg(II) ions in the polymers.

### Evaluation of porogen factors for the Hg(II) removal

The effects of different types and amounts of porogen in the IIPMs preparation (as stated in Table 1) were evaluated. Firstly, 100 mg of every IIPM prepared was conditioned with 10 mL of deionized water and 5 mL of methanol, successively. Then, 100 mL of Hg(II) solution (10 μg/L) was passed through the IIPMs at room temperature condition. Following this, the treated Hg(II) solution was collected for determining the concentration of Hg(II) using mercury analyser model Perkin Elmer FIMS 400. The mercury analyser and accessories were controlled using the Perkin Elmer Syngistix for AA system software. The operating parameters were the wavelength of Hg(II):253.7 nm, signal measurement: peak height and read time sample: 20 sec. The amount of Hg(II) adsorption was calculated using the following equation: $q\;=\;\frac{\left(Ci-Cf\right)\;X\;V}{M}$

Where q (μg/g) is the amount of total adsorption of Hg(II), C_i_ and C_f_ are the initial and final concentrations of Hg(II) ions in the solution (μg/L), v (L) is the total volume of solution and M (g) is the mass of IIPMs.

### Characterization of IIPs

The functional groups and types of bonds present in the samples were determined using the FTIR spectroscopy Model-100 series by Perkin Elmer (CT, USA). The technique used for sample preparation was the universal attenuated total reflectance (UATR). TGA analyses were performed using a Metler Toledo TGA/SDTA 851e model (Metler Toledo, Greifensee, Switzerland) controlled by STARe software version 10.00. The morphologies of samples were performed using FESEM instrument (JEOL-JSM-7600F model, Tokyo, Japan). The surface area and the total pore volume of the prepared IIPs were determined by using N_2_ adsorption-desorption method, BET apparatus (Autosorb-1 instrument controlled by Quantachrome AS1Win software).

## Results and discussion

### Effect of porogens for the preparation of IIPMs

Porogens play an important role for improving the porosity and water permeability of IIPMs. Here, the effect of different types and amounts of porogen in the IIPMs preparation were discussed in this study. Table 2 shows various types of IIPMs prepared using a different amount of porogen (methanol or acetonitrile). Based on Table 2, the preparation of various types of IIPMs was successful with the total yield for all the IIPMs were around 11.642–14.162 g. The porogens added in those polymers simply evaporates during the polymerization and left as pores after the completion of polymerization, and thus the mass of polymers obtained is not affected by the amount of porogen. According to the physical observations for all types of IIPMs, the hardness of IIPMs decreased with the increasing amount of porogen. The results further support that the porosity of IIPMs depends on the amount of porogen added to the polymers.

**Table 2:** Different types of IIPMs and their physical properties.

Type of IIPMs	Porogen type (mL)		Total volume before polymerization (mL)	Mass of IIPMs obtained (g)	Percentage (%) yield
	CH_3_OH	CH_3_CN			
IIPM-1	-	-	11.86	11.857	61
IIPM-2	5.0	-	16.86	13.479	70
IIPM-3	10.0	-	21.86	13.548	70
IIPM-4	20.0	-	31.86	12.458	64
IIPM-5	30.0	-	41.86	14.054	73
IIPM-6	-	5.0	16.86	11.642	60
IIPM-7	-	10.0	21.86	12.853	66
IIPM-8	-	20.0	31.86	14.013	72
IIPM-9	-	30.0	41.86	14.162	73

### Effect of porogens for the removal of Hg(II)ions

**Fig 1:**
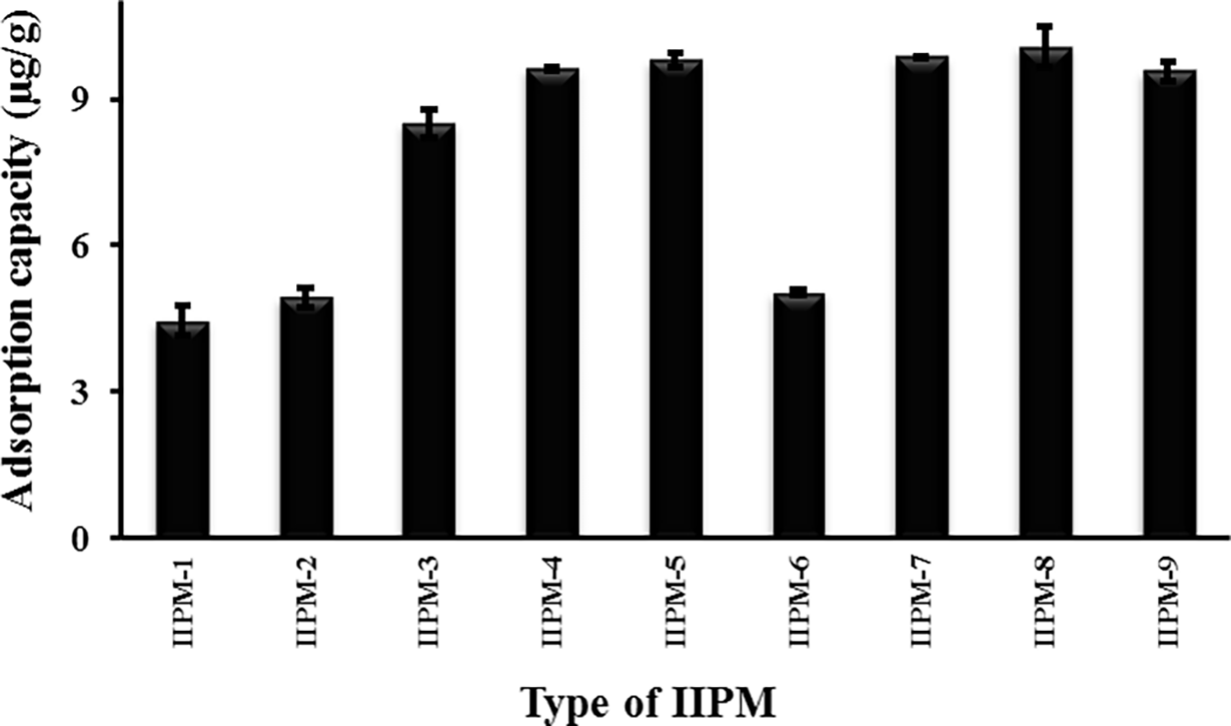
Effect of porogens for the removal of Hg(II); experimental conditions: 1.0 g/L adsorbent dose, 10 μL initial concentration of Hg(II), pH 4.7.

The pre-polymerization solutions with different volumes of porogen (methanol and acetonitrile) were added to prepare the monolithic polymers. The adsorption capacities of Hg(II) by different types of IIPMs are given in Fig 1. The graph shows that the adsorption capacities for IIPM-1, IIPM-2, and IIPM-6 were 4.446, 4.928, and 5.024 μg/g respectively. These values indicate that the adsorption capacities of Hg(II) were low when the porogen volume was less than 10 mL because more recognition sites were embedded deeply in the polymer matrix and became less accessible for the target ions [15]. Furthermore, these IIPMs have low porosity and water permeability, thus, its tend to decrease the adsorption of Hg(II) ions into the imprinting sites.

Meanwhile, the adsorption capacities of IIPM-3, IIPM-4, IIPM-5, IIPM-7, IIPM-8, and IIPM-9 were 8.510, 9.892, 9.738, 10.074, 9.796, and 9.569 μg/g respectively. These values indicate that the IIPMs with greater than or equal to 10 mL porogen contain good adsorption capacity due to high porosity and good water permeability of adsorbent. The characteristics of the monolithic polymers also are important factors for choosing the best IIPMs. From the observations, the IIPM-8 was selected as the best IIPM as compared to the other IIPMs in terms of maintaining the good physical properties, which is not too brittle, not too hard and easy to cut. For the IIPMs (IIPM-3 and IIPM-7) added with 10 mL of porogen are not selected because its structure is too hard and difficult to cut. Also, the IIPMs (IIPM-5 and IIPM-9) added with 30 mL of porogen are not selected because its structure is too brittle and easy to crack on getting contact with water.

### Scale up for the preparation of IIPs

Scaling up is a process to migrate the amounts of reagents from the lab-scale to the pilot plant-scale or commercial scale. The scale up for the preparation of IIPP and IIPM-8 has been successfully carried out up to 200 times as shown in Table 3. The mass obtained for both polymers increased consistently with an increase of total volume of the mixture of polymer solution (template ion, ligand, monomer, cross-linker, initiator and porogen). The comparison for the scaling up preparation between IIPP and IIPM-8 shows that the IIPP has low yield as compared to IIPM-8 because the IIPP needs to be crushed, ground and sieved into fine particles which caused the problems like more particles loss. This result further provides the evidence for the applicability of IIPM-8 towards the commercial/industrial scales as it is easy to prepare, very economic and no wastage of product.

**Table 3:** List of scale-up for the IIPP and IIPM-8 preparation.

Scale up (times)	Mass of polymers obtained (g)		Percentage of yield (%)	
	IIPP	IIPM-8	IIPP	IIPM-8
1	6.050	14.013	31	72
2	18.578	30.114	48	78
5	41.008	60.931	42	63
10	78.912	190.579	41	99
20	153.714	278.691	40	72
50	412.110	763.670	43	79
100	844.865	2151.77	44	111
200	1663.630	3552.600	43	92

### Characterization of different IIPs

#### FTIR analysis

**Fig 2:**
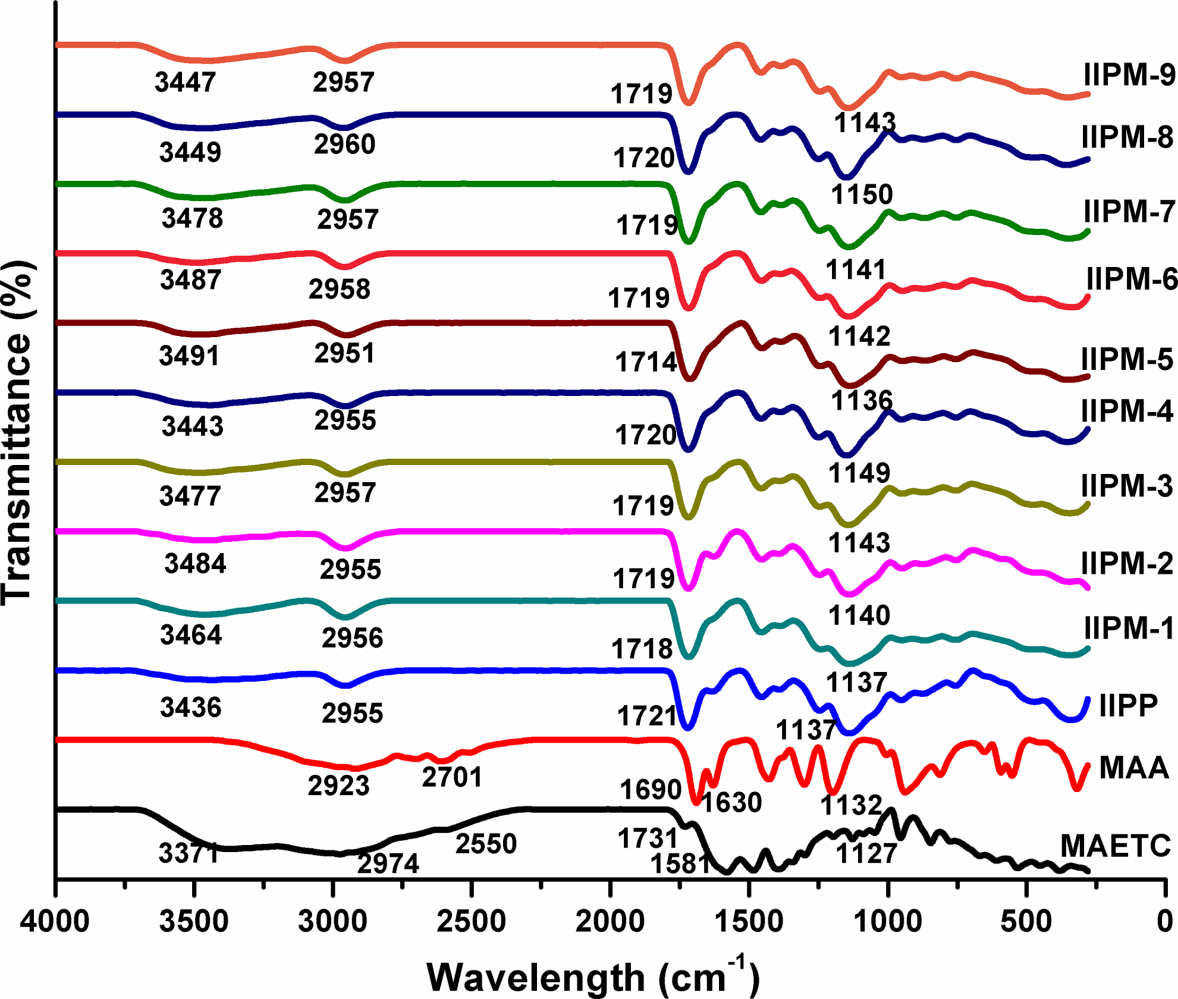
FTIR spectra for MAETC, MAA, IIPP and various types of IIPMs.

Fig 2 shows the FTIR spectra of MAA (monomer), MAETC (ligand), IIPP and various types of IIPMs. Based on Fig 2, all the spectrums for various types of IIPs (IIPP, IIPM-1, IIPM-2, IIPM-3, IIPM-4, IIPM-5, IIPM-6, IIPM-7, IIPM-8, and IIPM-9) showed near to similar absorbance wavelength owing to all polymers have a similar backbone and functional group. From these spectra, the medium intensity of N-H stretching vibrations from the MAETC compound can be observed for MAETC spectrum at 3371 cm^-1^ and all the IIPs in the range 3436–3491 cm^-1^. The broad O-H stretch peak showed for MAA compound can be observed at 2923 cm^-1^ and all the IIPs in the range 2951–2960 cm^-1^. However, the intensity of O-H stretch peak for IIPs gave smaller peak as compared to MAA due to the formation of an interaction of hydrogen bonding between monomer-MAA and MAETC compounds had occurred during the polymerization process. The frequency of N-H stretch and O-H stretch for IIPs shifted to higher frequency as compared to MAETC and MAA. This is due to the indication of relative strength of hydrogen bonding interaction getting occurred between the amine group (ligand) and carboxyl group (monomer) in the polymers. The sp^2^ and sp^3^ C-H stretching bands appeared for MAETC and MAA compound at 2974 and 2701 cm^-1^ respectively. Unfortunately, these bands are not appeared for all the IIPs because they overlap with O-H stretch peak, stronger adsorptions which occur in this region.

The FTIR spectrum of MAETC has shown the characteristic absorbance peak at 2550 cm^-1^, which can be ascribed to S-H (thiol group) vibrations. However, S-H peak for all IIPs disappeared in its spectra because the sulfhydryl groups could donate a lone pair of electrons to the empty orbit of Hg(II) ions alone. This confirmed that the formation of Hg-MAETC complex has occurred. All of the spectra showed of C = O and C-O stretching band for MAA, MAETC and all of the IIPs which are in the range 1690–1731 cm^-1^ and 1132–1150 cm^-1^ respectively. A stronger and sharper peak can be observed for all IIPs compared to MAA and MAETC. In the spectrum of MAA and MAETC, the C = C stretching band was determined at the absorbance of 1630 and 1581 cm^-1^ which were absent in all IIPs due to the occurrence of complete polymerization. Based on these results, we can confirm that the polymerization and imprinting process have successfully occurred for all the IIPs.

#### TGA study

**Fig 3:**
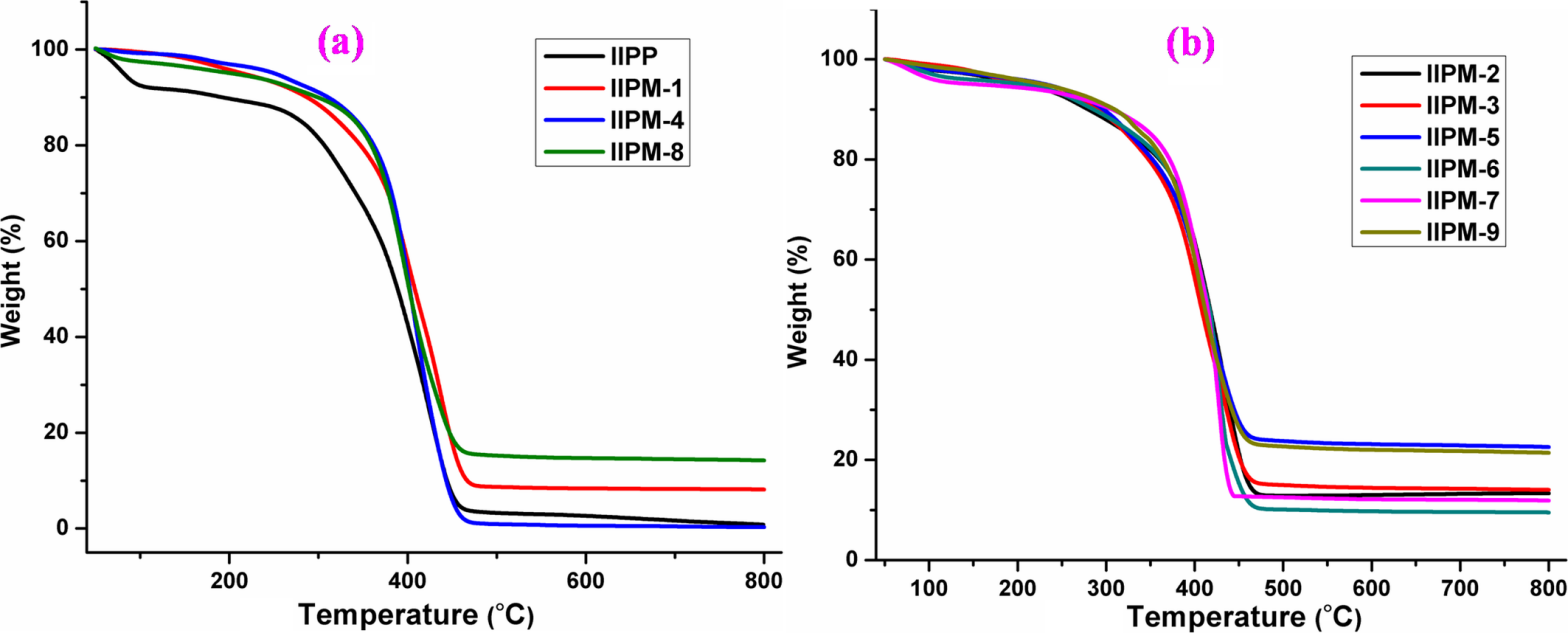
(a) TGA thermograms for IIPP, IIPM-1, IIPM-4 and IIPM-8 and (b) TGA thermograms for IIPM-2, IIPM-3, IIPM-5, IIPM-6, IIPM-7 and IIPM-9.

TGA is very important to show how stable is an imprinted polymer at elevated temperature conditions. The TGA thermograms shown in Fig 3(A) for IIPP, IIPM-1, IIPM-4 and IIPM-8 have a similar kind of degradation pattern. The initial decomposition (T_i_) of IIPP and IIPM-1 were 112 and 229°C and final decomposition temperature (T_f_) of IIPP and IIPM-1 were 517 and 491°C respectively. This indicates that the IIPM-1 started to decompose at the higher temperature as compared to the IIPP because more energy needed to break the polymers in monoliths form as compared to the polymers in particles form. Meanwhile, the T_i_ for IIPM-4 and IIPM-8 (same amount of porogen) were 206 and 194°C and T_f_ were 498 and 491°C respectively. This data shows that the IIPM-8 (binary porogen) has lower thermal stability as compared to IIPM-4 (single porogen) owing to IIPM-8 has higher porosity and surface area compared to IIPM-4 (the porosity and surface area of the polymers are discussed detailed in the following sections 3.4.3 and 3.4.5). Further, the Fig 3(B) shows that the TGA thermograms for various types IIPMs with porogen (IIPM-2, IIPM-3, IIPM-5, IIPM-6, IIPM-7 and IIPM-9) which are T_i_ and T_f_ for all IIPMs were in the range of 180 and 219°C and 424 and 570°C respectively. Based on (Fig 3A and 3B), it can be indicated that the high porogen presence in the polymers reduces the thermal stability of the compounds. For instance, the IIPM-1 (start degrade at 229°C) has higher thermal stability as compared to IIPMs prepared with high porogen (start degrade in the range 180 and 219°C). This is due to the fact that the IIPMs prepared with high porogen have high porosity and surface area, and thus they tend to have low thermal stability. However, IIPMs with high porogen still have high thermal stability as compared to the IIPP.

#### Morphological analysis

**Fig 4:**
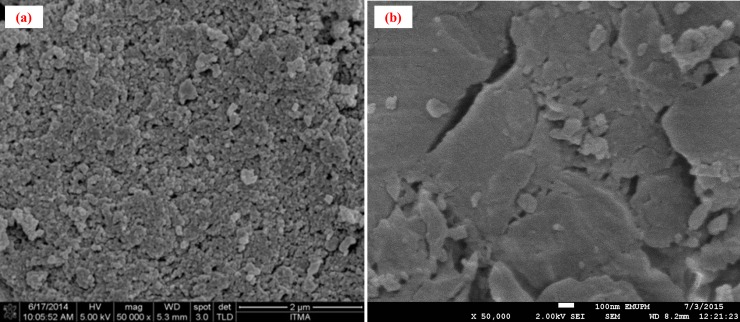
**Morphology of (a) IIPP and (b) IIPM-1 under the 50,000 magnification**.

The surface morphology by means of FESEM analysis for the IIPP and IIPM-1 (polymers prepared with the same method and amount of reagent but different physical form) under the magnifications 50,000 are shown in Fig 4. From the figure, the images have shown appreciable differences in the morphology of the polymers. The IIPP has more globular, aggregated, porous and rough morphology as compared to the IIPM-1 which is more smoother, packed, low porous and very dense with minimum pores and spaces like morphology. Due to the weakness of the IIPM-1, the porogen was applied in monolithic polymer preparation to improve the porosity of IIPMs and thus have enhanced the selective removal of Hg(II) ions.

**Fig 5:**
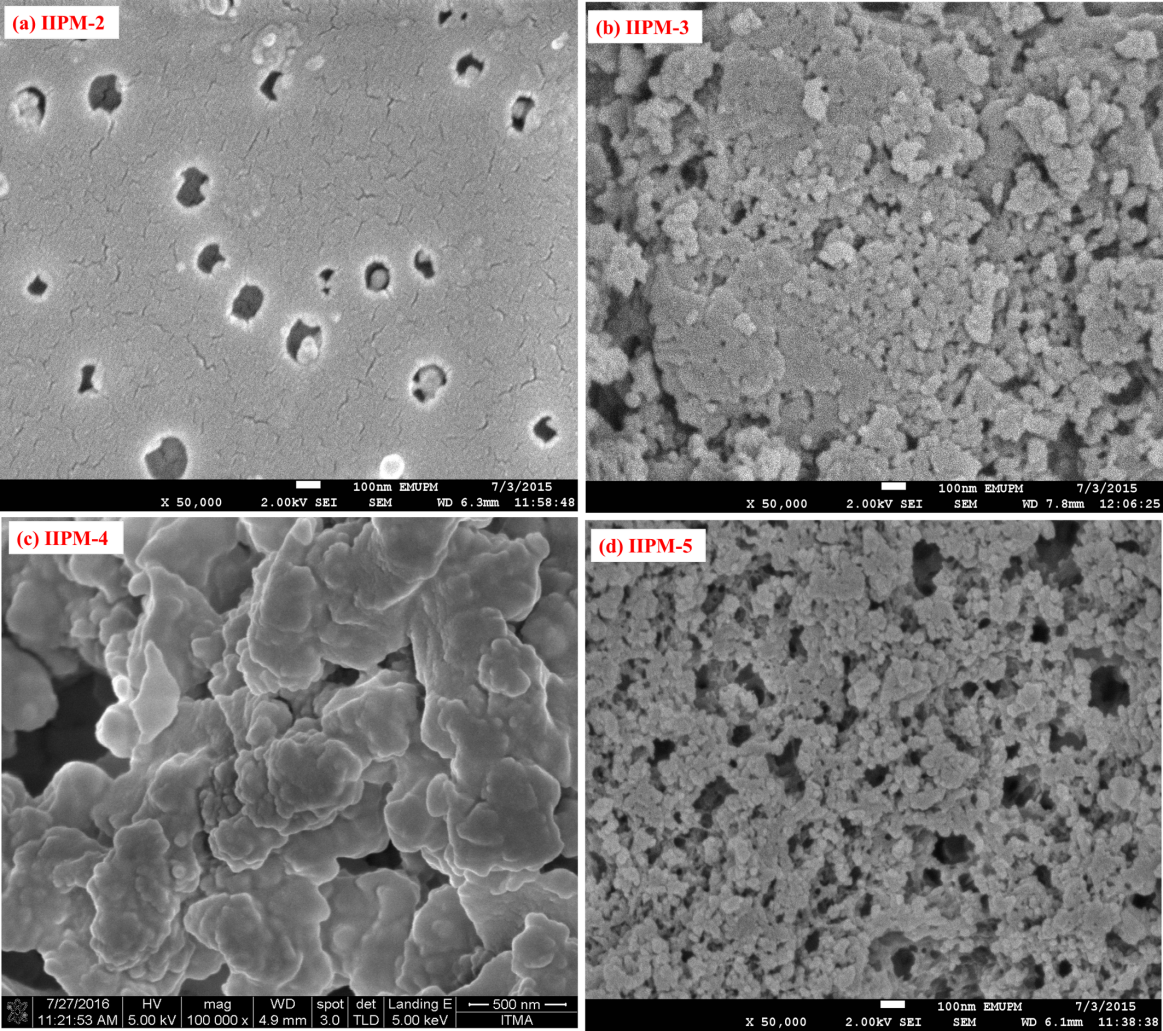
**Morphology of the polymers prepared using single porogen (a) IIPM-2, (b) IIPM-3, (c) IIPM-4 and (d) IIPM-5 under the magnification 50,000**.

**Fig 6:**
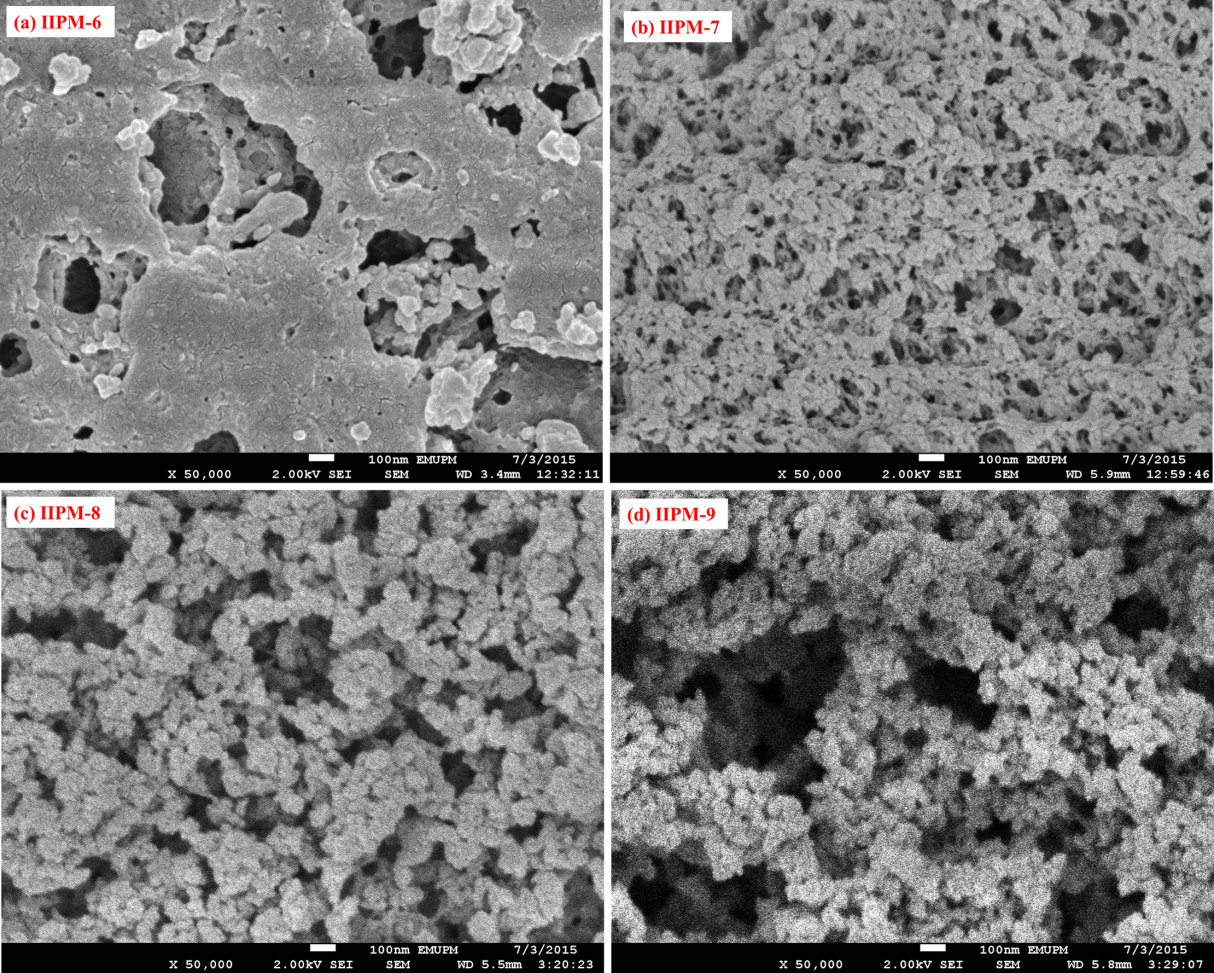
**Morphology of the polymers prepared in the presence of binary porogen, (a) IIPM-6, (b) IIPM-7, (c) IIPM-8 and (d) IIPM-9 under the magnification 50,000**.

An appropriate amount of porogen is very important to produce pores, in order to assure good flow-through properties of the resulting IIPMs. The IIPMs prepared with different types or porogen amounts may show different morphologies due to a different solvating power of the porogen towards the polymer chain [16]. Once the polymerization gets completed, the porogen molecules simply evaporate from their sites and so the leftover space originally occupied by the porogen remains as pores [14]. The surface morphology FESEM images of the various types of IIPMs when prepared in the presence of single porogen are shown in (Fig 5A–5D), while the binary porogen presence is shown in (Fig 6A–6D) (magnifications 50,000). According to the Fig 5 and Fig 6, the images have shown the globular, aggregated, porous and open structure of network skeleton with large flow-through pores and were achieved by increasing the amount of porogen. The IIPMs (IIPM-3, IIPM-4, IIPM-5, IIPM-7, IIPM-8 and IIPM-9) prepared with higher volume (more than 10 mL) of porogen have a larger porosity and good permeability as compared to IIPMs (IIPM-1, IIPM-2 and IIPM-6) prepared with lower volume (less than 10 mL). The higher volume (more than 10 mL) of porogen indicates that it could provide flow paths through the IIPMs and would be beneficial for the adsorption and desorption of Hg(II) ions. Other than that, the FESEM images also show that the IIPMs prepared with binary porogen are better than the IIPMs prepared with single porogen due to more porous and the excellent permeability of IIPMs. The observation of such increased porous effects in the presence of binary porogens can be linked to the differences in the amount of total space occupied by the porogen elements when adsorbed at the polymer site. Since the two porogens (CH_3_OH and CH_3_CN) with their differences in the total size and surface electrochemical effects has the ability to form the pores of varying size and volumes as compared to the single porogen of any amount. The use of a single porogen has a limited adsorption capacity onto the polymer base as against the binary porogen due to the improvement in the surface electron transfer and solubility effects which further governs the amount and stability of adsorption [17].

#### Surface area and porosity

The porosity for IIPP and all types of IIPMs were investigated by N_2_ adsorption-desorption measurements. The BET method was applied to determine the specific surface area and the Barret Joyner Halenda (BJH) method for the pore diameter estimation. The summary of data on the surface area, pore volume, average pore diameter, and isotherm type are listed in Table 4. The BET specific surface area, total pore volume and average pore diameter for IIPP and all of the IIPMs were around 6.493–183.069 m^2^/g, 0.023–0.833 cc/g and 3.050–9.708 nm respectively. All polymers showed typical “Type IV” nitrogen adsorption-desorption isotherm, which is in agreement with the capillary condensation that takes place in the mesoporous materials. Based on Table 4, the IIPP (55.970 m^2^ /g) has high surface area as compared to IIPM-1 (6.493 m^2^/g); however, the porogens added to those polymers were able to improve the surface area of the IIPMs in the range 14.435–183.069 m^2^/g. On the other hand, the table also indicates that the IIPMs added with binary porogen (in the range 114.156–183.069 m^2^/g) have exhibited a high surface area as compared to the IIPMs added with single porogen (in the range 14.435–61.192 m^2^/g). The observation of such result can be linked to the presence of methanol solvent where this as a polar porogen and offers a good solubility to the template ions, but may disturb the ionic interaction between the ligand and Hg(II) ions. Further, acetonitrile as an aprotic polar solvent can produce large pores and high surface area which in most cases may be unstable and does not support for the stability of Hg(II) ions inside the pores. For this reason, a mixture of porogens (binary porogen) with a suitable ratio is adopted for tuning the solubility, polarity and ability to generate the stable pores with improved surface areas of monolithic polymers [11,18]. From the table, it is clear that the IIPMs formed in the presence of a binary mixture are with higher surface areas as compared to the ones that were prepared using a single porogen. We found that the IIPMs-6, -7, -8 and -9 are formed with higher surface areas (above 110 m^2^/g) as against the IIPMs-1, -2, -3, -4 and -5 (surface areas below 60 m^2^/g). Also, in the binary porogen mixture, the IIPMs of higher surface areas are shaped with low pore volume and associated low pore diameter as against the single porogen which is supporting for the generation of low surface area and high pore volume/diameter. The formation of high surface area with less pore size diameter/volume means that more number of pores are forming and in a similar way, less surface area with high pore size diameter/volume is an indication for a less number of pores. The observation of such result can be attributed to the fact that the presence of two different porogens (CH_3_OH and CH_3_CN) in the reaction mixture are supporting for the formation of more number of pores with less size. However, a single porogen presence is encouraging for the generation of larger sized pores in a very less number and that is what making here to see the less surface area. These results can further serve as a decision making factor for the generation of pore sizes or surface areas to be in low or higher amounts by means of carrying the reactions in the presence of single or binary porogens. In addition, it can be noted from the table that the IIPM-6 and IIPM-7 have higher surface areas as compared to others, but their structural morphologies indicate that they are too hard and difficult to cut during the cutting process and so limit their selection as the best for Hg(II) adsorption. Similarly, the IIPM-9 material too is considered to be not so good adsorbent due to its too brittle structure and easy cracking nature. Although the IIPM-8 has not maintained a very high surface area as compared to IIPM-6, IIPM-7, and IIPM-9, by considering the structural morphology aspects we selected this as the best one for the adsorption. In general, not just the surface area is the important factor for determining the best IIPM, but other aspects like stability, softness, resistance etc also needs to be considered for selecting the monolithic polymers.

**Table 4:** Data of surface area, pore volume, average pore diameter, and isotherm type for different types of IIPs.

Type of IIPs	Surface area (m^2^/g)	Total pore volume (cc/g)	Average pore diameter (nm)	Isotherm type
Single porogen (CH_3_OH)				
IIPP	55.970	0.283	6.595	IV
IIPM-1	6.493	0.023	9.708	IV
IIPM-2	39.675	0.123	3.072	IV
IIPM-3	61.192	0.368	5.617	IV
IIPM-4	14.435	0.100	3.449	IV
IIPM-5	67.311	0.195	5.653	IV
Binary porogen (mixture of CH_3_OH and CH_3_CN)				
IIPM-6	134.176	0.833	3.050	IV
IIPM-7	183.069	0.716	3.146	IV
IIPM-8	114.156	0.241	3.822	IV
IIPM-9	118.160	0.678	4.905	IV

## Conclusion

In conclusion, we indicate that the porous IIPMs were prepared using the molding process in the drinking straw and successfully applied for the removal of Hg(II) ions from aqueous solutions. The prepared IIPP and all of the IIPMs were characterized successfully using FTIR, TGA, FESEM and BET analysis. The morphologies for the IIPMs prepared with higher volumes of porogen were more porous as compared to the lower volume porogens. The IIPMs added with binary porogens (CH_3_OH and CH_3_CN) have a high surface area compared to IIPMs added with single porogen. From the comparison and analysis of different IIPMs, the IIM-8 was selected as the best of IIPMs owing to its good Hg(II) adsorption, surface area, porosity and the physical properties. The scaling up preparation between IIPP and IIPM-8 shows that the IIPP has low yield as compared to the IIPM-8 because the IIPP needs to be crushed, ground and sieved which in general results for the issues like more particles loss. The data further demonstrates that the IIPMs adjusted porogen revealed much high permeability, adsorption capacity, porosity and surface area. Furthermore, the prepared IIPMs (IIM-8) can be able to overcome the weaknesses and disadvantages of IIPP packed columns on an industrial scale for the selective adsorption of Hg(II) ions from waste water samples.
