# Correction: Evaluation of porogen factors for the preparation of ion imprinted polymer monoliths used in mercury removal

**DOI:** 10.1371/journal.pone.0208242

**Published:** 2018-11-26

**Authors:** The PLOS ONE Staff

There is an error in affiliation 3 for authors Faruq Mohammad and Hamad A. Al-lohedan. Affiliation 3 should be: Surfactant Research Chair, Department of Chemistry, College of Science, King Saud University, Riyadh, Kingdom of Saudi Arabia. The publisher apologizes for the error.
